# Comparative Study of Biotin and Hydroxyapatite on Biological Properties of Composite Coating

**DOI:** 10.1155/2022/8802111

**Published:** 2022-09-26

**Authors:** Qahtan A. Hamad, Fatimah J. Al-Hasani, Noor K. Faheed

**Affiliations:** ^1^Materials Engineering Department, University of Technology, Baghdad, Iraq; ^2^Department of Petroleum Engineering, University of Misan, College of Engineering, Misan, Iraq

## Abstract

The ultimate goal of using biomaterials is to improve human health by restoring the function of natural living tissues and organs in the body. The present work aims to modify the composite coating layer properties by using two different types of bioactive reinforcing materials (biotin and hydroxyapatite) particles in different percentages (5% and 10%). Coatings were applied onto commercially pure Ti, SS 316 L, and SS 304 substrates by the dip-coating method. Characterization of samples includes microstructure observation by field emission scanning electron microscopy (FE-SEM), contact angle measurement (wettability), and MTT. The results show the addition of metallic particles (bioparticles) (hydroxyapatite particles, biotin) at 5 Vol. % improved the whole properties of composite materials. Using different particles' scale size aids to enhance the combinations in the alginate matrix producing a dual effect on composite film properties. In addition, the inclusion of metallic particles has to increase the wettability by reducing the contact angle. At the same time, MTT graphs revealed that after 3 days of exposure in MG-63 cells, 316 L SS alloys' surface following pack adhesion became more active.

## 1. Introduction

The biomaterial has not been around for a very long time. Nonbiological compounds were first introduced into the human body thousands of years ago [1]. The majority of biomaterials are already accessible for use in the development of these materials, either individually or in combination. These materials have various atomic configurations, resulting in a wide range of structural, physical, chemical, and mechanical properties, as well as a wide range of potential applications in the body [2, 3]. Because human plasma is salty, the metal used to heal the broken bones corrodes, resulting in an inflammatory reaction, tissue colonization, and allergic reactions in the individuals. Passive metals like 316 L stainless steel, titanium alloys, and cobalt alloys are preferable for orthopedic implants. Regionalized corrosion of pitting, fissures, and erosion persists nevertheless since the Cl ion in human plasma can enter the passive layer at local faults [4]. Along with osteogenesis, suitable mechanical qualities, super corrosion, and erosion resistance, biocompatibility is one of the necessary conditions that metal biomaterials for medical purposes must meet [5, 6]. Furthermore, given their widespread utilization in biotechnology, medicine, dentistry, and biotechnology, biomaterials should be safe and should not cause inflammation or hypersensitive reactions [1]. Most biomaterials are composite coatings made up of different materials with different atomic configurations and different structural, physical, chemical, and mechanical characteristics [3]. In comparison to other metallic biomaterials, titanium and its alloys fulfill these parameters [7]. Titanium alloys have high biocompatibility and low dissolution in body fluids, making them acceptable and harmless within the body, as well as providing excellent bioadhesion [8]. Coatings are among the most utilized approaches in biomedical disciplines to enhance the bone density of Ti and Ti alloys because of their well-documented advantages, such as physiologically active coatings [9]. The most popular biomedical metals for prosthetic knee joints, joint stocks and dentures, dental implants, root metal stents, etc., are titanium and titanium alloys because of their outstanding biocompatibility, resistance to corrosion, mechanical characteristics, and process effectiveness [10]. Medical implants, including orthopedic pins, screws, and knee implants, are commonly made of stainless steel (grade 316 L). As a result, employing 316 stainless steel antibacterial coatings to reduce the risk of interactive infections and surgical complications would be beneficial [11, 12]. Stainless steel alloys have a minimal price, are readily available, have good training qualities, are biocompatible, and have high strength [6]. Because it has a stretched protecting layer that resists corrosion, stainless steel (316 L) has high biocompatibility [13]. 304 stainless steel, a less costly and more widespread version, is particularly corrosion-resistant steel due to the creation of passivity, and it has a wide spectrum of uses in foodstuff containers, oxidizing acid storage tanks, kitchenware, and medical tools. However, because of its lower corrosion resistance in human bodily fluid, it is not favored as an implant material above the other materials indicated above [4]. Alginate is regarded as safe for biological devices because it is utilized in food research for human ingesting. Alginate is also biodegradable because it dissolves gradually in the body when cross-linking means in the alginate discharge and interchange process interact with monovalent cations prevalent in human fluids [14]. Alginate recently blended with several materials presenting alginate composites providing the needs of other biomedical requirements and improving soft tissue creation and repair [15]. Blending different kinds of materials, like bioglass, ceramics, inorganic nanoparticles, and inorganic carbon-based materials, have also been investigated [16].

In 2009, Sanpo et al. [17] studied the antimicrobial behavior of CS-Cu composite coatings against *E*. *coli* (DH5A). The results showed that the effects of all composite powders increased to the proportion of the effects of CS-Cu powder because of the cold aerosol. The characterization and analysis of the phase of the microstructure of the raw material and the deposition coating were performed using field emission electron microscopy (FESEM)/EDX and Fourier transform infrared (FTIR) spectroscopy. In 2013, Carneiro et al. [18] showed that the interactions of organic molecules with 2-mercaptobenzotiazole (MBT) release corrosion inhibitors and polymer coating systems for the protection of the (II) aluminum alloy AA2024. They were able to prepare a CTS-MBT composite and use it as an “intelligent” pigment in a good barrier for high-performance applications. In 2017, Xihua et al. [19] studied the efficacy of the corrosion of the mild steel biotinic drug in 15% HCl acid with weight loss and electrochemical methods. Immersion time, temperature, activation energy, inhibitors, and concentration were studied. Polarization studies explored the mixed inhibitors of biotin. Biotin adsorption is typically followed by the isothermia of Langmua adsorption. Studies showed steel hydrophobicity with inhibitors prevents the formation of films on metallic surfaces with reduced corrosion rates. In 2020, Ahmed et al. [20] used electrophoresis techniques to improve the bone conductivity of 316 L stainless steel and its corrosion resistance. The complex coatings of HA-Zein are deposited. Improvement in performance in biological environments and adhesion of high-capacity training in simulated body fluid (SBF). Issa et al. [21] used electrostatic deposition to create a thin poly (methyl methacrylate) compound coating layer from various bioceramics as reinforcing materials. The resulting coating layers were homogenous, showed no cracking, and had mechanical properties [21].

One approach to increasing osseointegration and helping protect the implant from any ions generated or assaulted by ions corroding is to coat the implant's metallic surface with a biocomposite coating that promotes the attachment and proliferation of bone cells. The current research seeks to investigate the use of dip-coating deposition to describe an alginate-based composite coating strengthened with biotin and hydroxyapatite and to improve the surface of commercially pure Ti alloy, 316 stainless steel, and 304 stainless steel.

## 2. Experimental Part

### 2.1. Fabrication of Testing Samples

As a substrate, economically pure Ti discs (Thommen Medical, Waldenburg, Switzerland; (2*∗*1*∗*1 mm) with (304 L and 316 L) stainless steel austenitic plate (20 mm in diameter and 1 mm in thickness) were utilized. The Ti plate's chemical composition was as follows: C has a 0.0075 percent ratio, H has a 0.014 percent ratio, and Ti is balancing. Following chemical elemental composition, the surface of the substrate was mechanically molded to 80°, 100°, and 150° to enhance the roughness of the surface. The substrate was then cleaned with deionized water and acetone before being dried. The dip-coating process was employed to create a composite coating film of alginate-based composites with various strengthened materials, as shown in Table 1.

For a pure alginate coating sample, the alginate powder was dissolved in diluted acetic acid to produce a milky solution. The solution is then put on a vigorous stirrer to achieve homogeneity and eradicate any remaining bubbles, and the pH is determined. By removing the NaOH solution, the pH was raised to 6.0. After 30 minutes in the alginate solution, the samples are covered with a thin layer of composite coating (hydroxyapatite and biotin) in the solution. An alginate-based composite coating (alginate + Nanosilver, alginate + biotin, or alginate + hydroxyapatite + biotin) is applied to the specimen.

Following coating, the coated samples were kept at ambient temperature once more before being kept in polyester containers. FESEM, MTT, and contact angle (wettability) assessment tests were employed to analyze the morphology of the coated specimens.

## 3. Methodology

**Figure 1:**
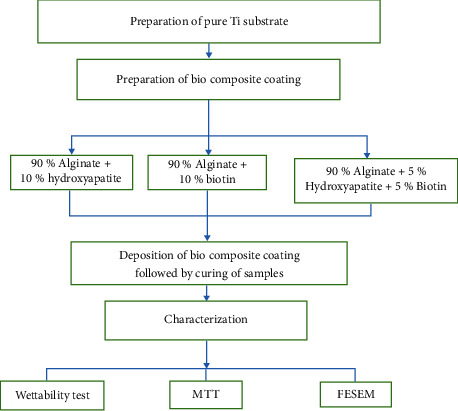
Composite and coating preparation procedure.

Composite and coating preparation procedure may be added in the form of a schematic diagram as shown in Figure 1.

### 3.1. Field Emission Scanning Electron Microscopy (FESEM)

The morphologic properties of all composite films were evaluated by field emission scanning electron microscopy (FESEM). Also, the microstructural observation of composite film samples was characterized by using (FESEM).

### 3.2. Contact Angle Measurement (Wettability)

The performance properties can be characterized by studying the wettability test to evaluate the water contact angle utilized to quantity surface hydrophilicity by assessing how much water droplet could feast on a surface.

### 3.3. MTT Assay (Cell Viability)

In vitro biocompatibility (MTT assay) was determined to evaluate the whole properties and biological behavior of the composite film to obtain a superior performance of implants when using the composite film as coating layer inside the body. Mitochondrial dehydrogenase activity (MTT assay) was used to measure the three-dimensional cell development of human MG-63 fibroblast cultures after 24, 48, and 72 hours of treatment.

## 4. Results and Discussion

### 4.1. Morphological Analysis (FESEM)

**Figure 2:**
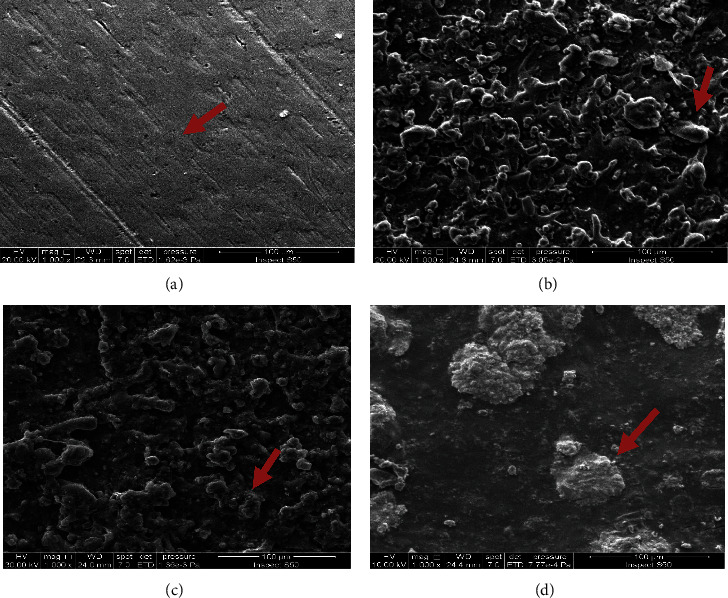
SEM images for (a) pure titanium, (b) hydroxyapatite particles in alginate matrix, (c) biotin in alginate matrix, and (d) biotin and hydroxyapatite in alginate matrix.

Microstructural observation of composite film samples was conducted using Field emission scanning electron microscopy (FE-SEM). Images show complete surface observation of samples, which clarifies the effect of metallic particle additions (hydroxyapatite, biotin) on the surface texture and roughness.  Figure 2(a) shows the microstructure observation of pure titanium without any addition. The homogenous and smooth surface texture of the Ti substrate was observed. The irregular nature of the inorganic component and the relatively inhomogeneous distribution of hydroxyapatite powder in the alginate matrix were observed. The addition of 10 vol.% of hydroxyapatite to alginate created a heterogeneous surface texture, and as a result, the morphology of the surface has completely changed, leading to an increase in the surface roughness, as mentioned in Figure 2(b). We noted that the surface was completely covered, and this indicates a high correlation between the base material and the reinforcing material and a gradually rough surface and morphological modification.  Figure 2(c) shows the microstructure image of the alginate matrix with 10 vol.% biotin additions. The surface texture was completely changed due to the particle's effect, with a possible decrease in surface roughness compared with the hydroxyapatite reinforcing composite film and high homogeneity between the alginate substrate and biotin as a reinforcing material due to the small particle size and the nature of the material. Therefore, the coating material became very compatible with the titanium surface. The addition of two different types of reinforcing particles (hydroxyapatite particles (HA) and biotin) added large changes in all surface textures, as shown in Figure 2(d). It was observed that the biotin precipitates at the surface of HA particles in addition to the alginate polymer matrix. Differences in particle size made the composite surface rougher with preferable homogeneity due to a high correlation of granular sizes between the reinforcement particles and their regular distribution in the substrate. Thus, mixing reinforcement materials of different types produces a high-quality coating material with good surface biological properties. Using different particle scale sizes aids in improving the combinations between them in the alginate matrix. The large surface area of biotin particles increases the attractive forces and facilitates biotin precipitation at the HA particle's surface, resulting in the production of a preferable combination of particulate reinforcing materials with a superior dual effect in improving the overall composite film properties [22, 23].

**Figure 3:**
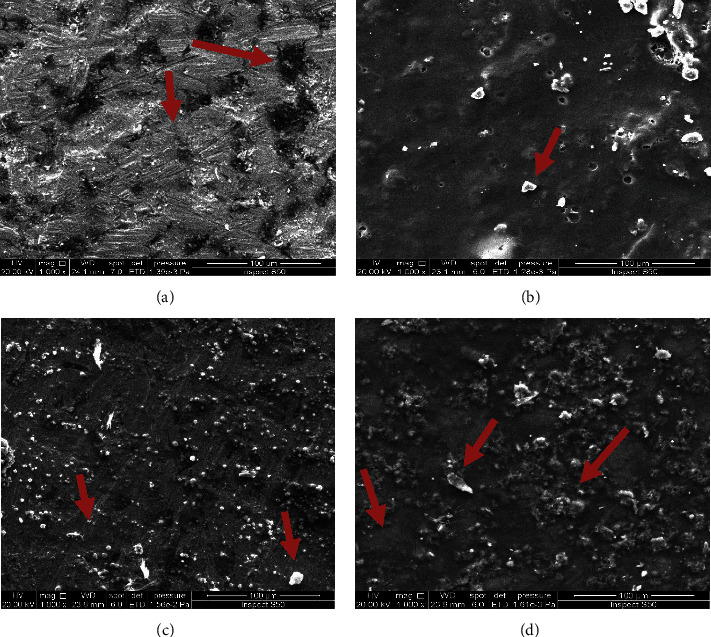
SEM image for (a) SS 316 L, (b) hydroxyapatite particles in alginate matrix, (c) biotin in alginate matrix, and (d) biotin and hydroxyapatite in alginate matrix.

Figure 3(a) shows the microstructure observation of 316 stainless steel without any addition. The homogenous and smooth surface texture with surface roughness more than the titanium sample was observed, and this roughness was the result of manufacturing processes. The irregular nature of the inorganic component and the relatively inhomogeneous distribution of hydroxyapatite powder in the alginate matrix were observed by the addition of 10 vol.% of hydroxyapatite to alginate, which created a heterogeneous surface texture, leading to an increase in the surface roughness as mentioned in Figure 3(b).  Figure 3(c) shows the microstructure image of the alginate matrix with 10 vol.% biotin additions. The surface texture was completely changed due to the particle's effect, with a possible decrease in surface roughness compared with the hydroxyapatite reinforcing composite film. Because of the high surface smoothness, biotin particles appeared clearly and evenly distributed over the base material. The addition of both (hydroxyapatite particles (HA) and biotin) presented large changes in all surface textures, as shown in Figure 3(d). It was observed that the biotin particles precipitated at the surface of HA particles in addition to the alginate polymer matrix, which has achieved high homogeneity and compatibility between the base material and the strengthening material, making the composite surface rougher. Using different types of reinforcing particle scale sizes aids in improving the combinations between them in the alginate matrix. The large surface area of biotin particles enhances the attractive forces and facilitates the precipitation of biotin at the HA particle's surface, resulting in the production of a preferable combination of particulate reinforcing materials that has a superior dual effect on the improvement of the whole composite film properties [23, 24].

**Figure 4:**
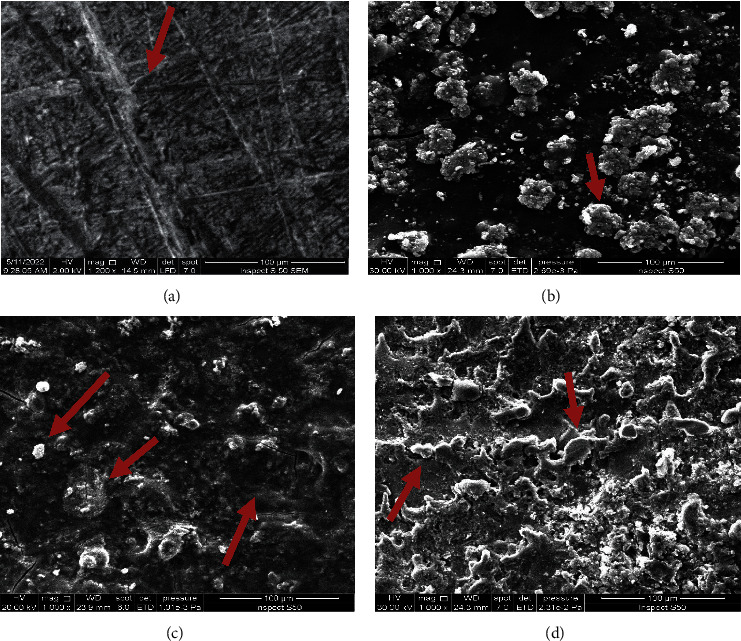
SEM image for (a) SS 304, (b) hydroxyapatite particles in alginate matrix, (c) biotin in alginate matrix, (d) biotin and hydroxyapatite in alginate matrix.

Figure 4(a) shows the microstructure observation of 304 stainless steel without any addition. The homogenous and smooth surface texture of the 304 L substrate was observed. Before the coating process, we noticed that the surface roughness observed was more than that of pure titanium and stainless steel 316 L, and this roughness is the result of smoothing and polishing processes of the sample. The irregular nature and the relatively nonuniform distribution of hydroxyapatite particles in the alginate matrix were observed with the inclusion of 10 vol.% of hydroxyapatite to alginate, which created a heterogeneous surface texture, as mentioned in Figure 4(b). These particles will induce a gradually rough surface and morphological modification, but the composites will keep their porous structure.  Figure 4(c) shows the microstructure image of the alginate matrix with 10 vol.% biotin additions. The surface texture was altered due to the particle's effect, with a possible decrease in surface roughness and an increase in the homogeneity of the surface. The addition of both (hydroxyapatite particles (HA) and biotin) presents noticeable alterations in surface textures, as shown in Figure 4(d). It was observed that using different particle scale sizes aids in improving the combinations between them in the alginate matrix. The large surface area of biotin particles enhances the attractive forces and facilitates the precipitation of biotin at the HA particle's surface, resulting in the production of a preferable combination of particulate reinforcing materials that has a superior dual effect on the improvement of the whole composite film properties [22].

### 4.2. Contact Angle Measurement (Wettability)

**Figure 5:**
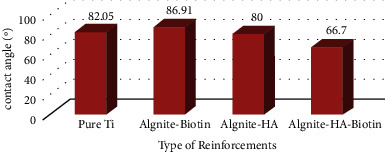
Contact angle results for alginate-based composite coatings (hydroxyapatite and biotin).

**Figure 6:**
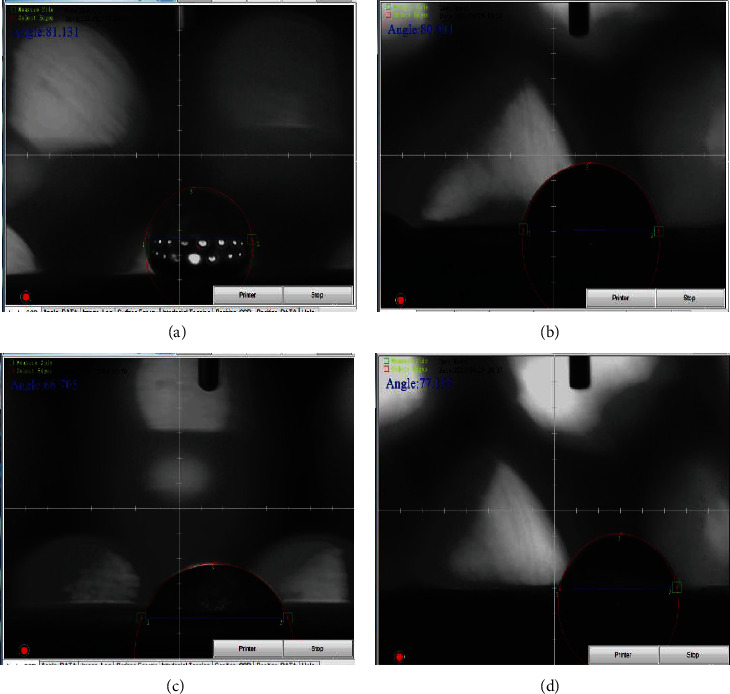
(a) Contact angle of pure titanium, (b) hydroxyapatite particles in alginate matrix, (c) biotin and hydroxyapatite in alginate matrix, and (d) biotin in alginate matrix.

Figure 5 depicts the contact angle readings of all specimens, explaining the impacts of multiple metallic particles and the wettability of composite material.  Figure 6(a) depicts images of the pure Ti sample's contact angle measurement. The surface showed a contact angle of roughly 82.05^o^, suggesting that it was hydrophilic. As shown in Figure 6(b), the addition of 10% hydroxyapatite to the alginate matrix reduces the contact angle to 80°. Because hydroxyapatite is hydrophilic, the addition of this quantity reduces the contact angle of composite materials and makes the surface highly hydrophilic.  Figure 6(c) shows the contact angle of the alginate matrix mixed with 10% biotin. The hydrophilicity of the surface rose with the inclusion of biotin, with a contact angle of 86.91°. This conduct was attributable to the reinforcement influence, which creates good and high bonding between the polymer matrix and nanoparticulate reinforcement, lowering roughness, and improving surface homogeneity. The addition of both metallic particles to the alginate polymer matrix results in a hydrophilic surface with a contact angle of 66.7°, as shown in Figure 6(d), due to the precipitation of biotin particles at the surface of (HA) particles in addition to the polymer matrix, which makes the surface of composites rougher with high energy [25, 26].

**Figure 7:**
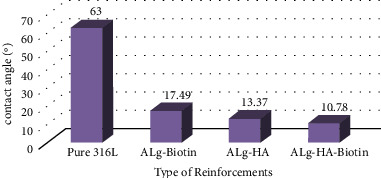
Contact angle results for alginate-based composite coatings (hydroxyapatite and biotin).

**Figure 8:**
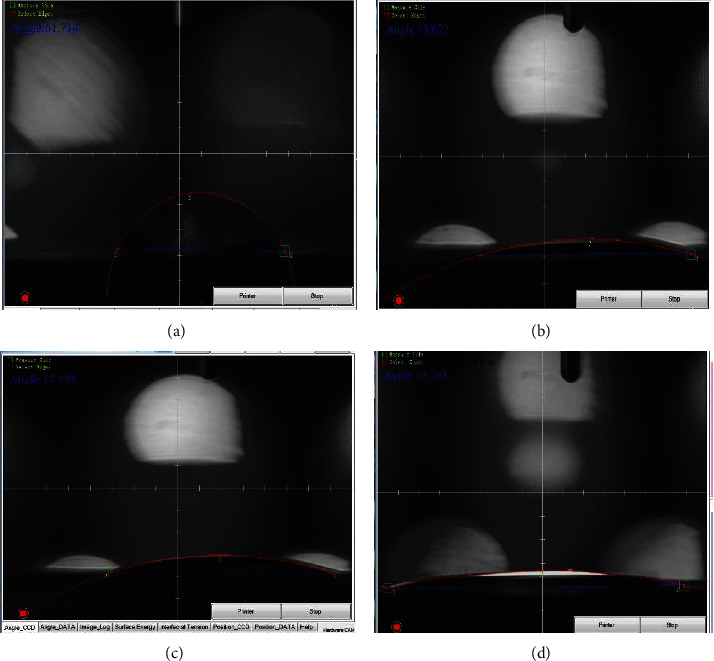
(a) Contact angle of SS 316 L., (b) hydroxyapatite particles in alginate matrix, (c) biotin in alginate matrix, and (d) biotin and hydroxyapatite particles in alginate matrix.

Figure 7 depicts the contact angle values of all specimens to define the impact of various metallic particles and the wettability of composite material.  Figure 8(a) depicts images of the pure 316 L sample's contact angle. The surface has a contact angle of roughly 63°, suggesting its hydrophilicity. As shown in Figure 8(b), the presence of 10% hydroxyapatite in the alginate matrix reduces the contact angle to 13.37°. Since hydroxyapatite is hydrophilic, the inclusion of this quantity reduces the contact angle of composite materials and makes the surface increasingly hydrophilic.  Figure 8(c) presents the contact angle of the alginate matrix with 10 vol.% biotin of 17.49°. From the figure, it is clear that the hydrophilicity of the surface decreased with the addition of biotin. This behavior was due to the reinforcement effect, which leads to good and high mechanical interlocking between the matrix and nanoparticulate reinforcement, thereby reducing the roughness and enhancing the surface homogeneity. The contribution of both metallic particles to the alginate polymer matrix results in a remarkable hydrophilic surface with a contact angle of 10.78°, as shown in Figure 8(d), due to the precipitation of biotin particles at the surface of (HA) particles in addition to the polymer matrix, which makes the surface of composites rougher with high energy [26, 27].

**Figure 9:**
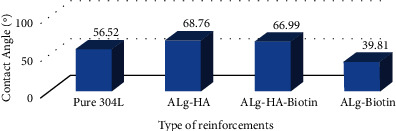
Contact angle results for alginate-based composite coatings (hydroxyapatite and biotin).

**Figure 10:**
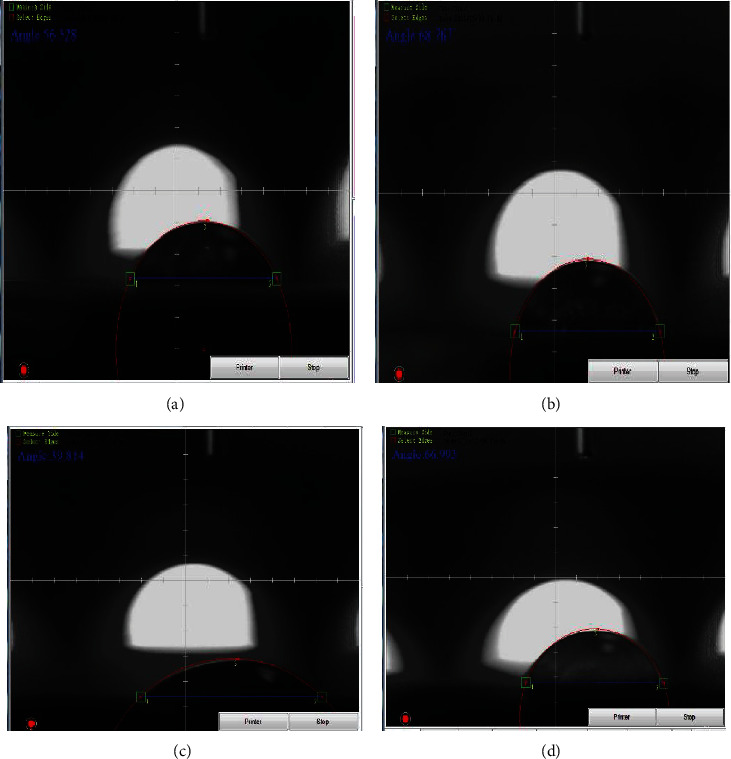
Contact angle for (a) SS 304, (b) hydroxyapatite particles in alginate matrix, (c) biotin in alginate matrix, and (d) biotin and hydroxyapatite in alginate matrix.

Figure 9 depicts the contact angle numbers of all specimens to interpret the influence of various metallic particles and the wettability of composite material.  Figure 10(a) depicts images of the pure 304 L sample's contact angle. The surface showed a contact angle of 56.52°, showing its hydrophilicity.  Figure 10(b) shows that the presence of 10% hydroxyapatite in the alginate matrix resulted in a contact angle of 68.76°. Because hydroxyapatite is hydrophilic, the presence of this quantity reduces the contact angle of composite materials and makes the surface particularly hydrophilic. As illustrated in Figure10(c) the contact angle of the alginate matrix with 10% biotin. It was evident from the figure that the hydrophilicity of the surface was reduced with the inclusion of biotin, with a contact angle of 39.81°. This attitude was attributable to the reinforcement impact, which allows for improved and high adhesion amid the matrix and nanoparticulate reinforcement, minimizing roughness and maximizing surface homogeneity. The combination of both metallic particles with the alginate polymer produces a more hydrophilic surface with a contact angle of 66.99°, as shown in Figure 10(d). The precipitation of biotin at the surface of HA particles, in addition to the matrix, causes the surface of composites to be rougher and more energy-efficient [28, 29].

### 4.3. MTT Assay (Cell Viability)

In general, the cells were plated in a 96-well tissue culture plate at a density of 104 cells per well and cultured for 24 hours in 100 L of DMEM/F12 mixed with 10% heat-inactivated fetal bovine serum (FBS). The culture media was transferred to a fresh serum-free culture medium, including serial dilutions of the specimen, and the cells were stored for 4 hours. The media was then charged with 100 L of fresh full media for a further 24 hours. The medium was substituted with 100 L of fresh medium containing MTT, yielding a total MTT content of 0.5 mg/ml, and the cells were stored for 4 hours at 37°C. The media was evacuated after 4 hours, the MTT formazan produced by living cells was added to 100 ml of DMSO, and the absorbance (570 nm) of each well was recorded by utilizing a microplate reader. The relative cell vitality (%) was calculated as [*A*] test/[*A*] control × 100, where [*A*] test and [*A*] control are the absorbance readings from the test treatment wells and control wells, respectively (control cells cultured in media without CDs). Data is provided as an average SD (*n* = 3). Three-dimensional environment mitochondrial dehydrogenase activity) was used to monitor cell growth in human MG-63 fibroblast cells over time (24, 48, 72 hours).

**Figure 11:**
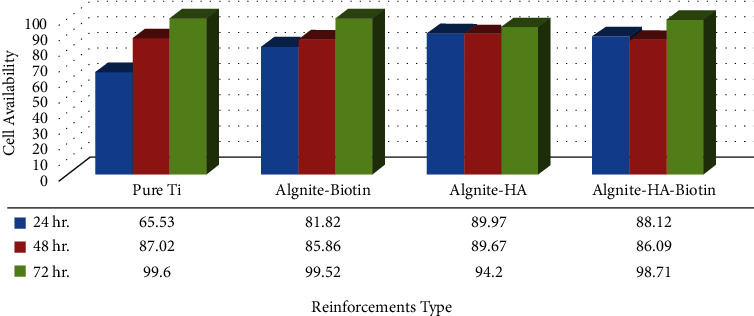
MTT graph of alginate in different reinforcing materials.

Figure 11 depicts the MTT graph of various reinforcing materials. Pure titanium demonstrated a high level of biocompatibility (cell viability) after 24 hours, 48 hours, and 72 hours of exposure. The introduction of active metallic particles as strengthening materials to the alginate matrix has a major impact on the composite's cell survival. Utilizing biotin nanoparticles to strengthen alginate leads to improved MG-63 cell proliferation at the surface, leading to increased cell viability in 24, 48, and 72 hour periods of exposure due to biotin's impact on lowering the rate of degradation. Furthermore, as shown in the figure, (Ti–alginate- hydroxyapatite) alloys showed an increase in cell viability with exposure time, showing that they have high biocompatibility and are suitable materials for implant fabrication. It also had no harmful impact on human cells, enabling the study of beneficial cell reactions to the alloy's surface. Because of the large changes in surface characteristics, it was clear that all samples' biological activity did not develop correctly in terms of cell viability. Because of variations in surface properties, the development of hydroxyapatite and biotin, which are essentially biomedical materials, did not increase surface bioactivity. Due to differences in surface properties, there was also a small difference in cell viability among alloys. As seen in the MTT plots, the investigation suggests that certain alloys have a considerable effect on cell viability [30, 31].

**Figure 12:**
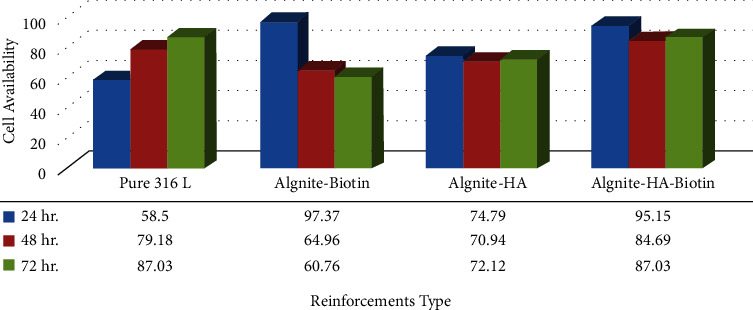
MTT graph of alginate in different reinforcing materials.

Figure 12 depicts the MTT columns of various strengthening materials. According to the MTT figure, at 24, 48, and 72 hours of exposure, pure 316 stainless steel demonstrated a high level of biocompatibility (cell viability). The incorporation of active metal particles as strengthening materials into the alginate matrix has a major impact on the composite's cell growth. Utilizing biotin reinforcing particles to strengthen alginate leads to improved MG-63 cell proliferation at the surface, resulting in greater cell viability in (24, 48, and 72) hour periods of exposure due to biotin's effect on lowering the degradation process. Furthermore, as shown in the figure, (316 L-alginate-hydroxyapatite) alloys displayed an increase in cell growth with exposure time, showing that they have excellent biocompatibility and are suitable materials for implant fabrication. It also had no harmful impacts on human cells, enabling the study of a beneficial response to the alloy's surface. Furthermore, cell viability exhibits no symptoms of aggression because of 316 stainless steel or other additions. Because of variations in surface properties, the development of hydroxyapatite and biotin, which are essentially biomedical materials, did not increase surface bioactivity [31].

**Figure 13:**
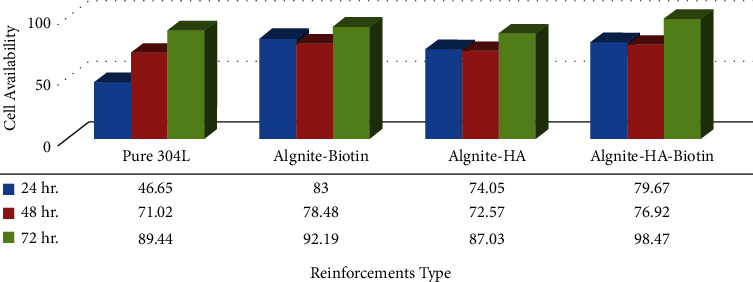
MTT graph of alginate in different reinforcing materials.

Figure 13 depicts the MTT results of various strengthening materials. According to the MTT figure, pure 304 stainless steel demonstrated a higher degree of biocompatibility (cell viability) after (24, 48, 72) hours of exposure periods. The introduction of active metallic particles as strengthening materials to the alginate matrix has a major impact on the composite's cell growth. Utilizing biotin particles to strengthen alginate leads to improved MG-63 cell proliferation at the surface, resulting in very high cell viability in (24, 48, and 72) hour exposure periods due to biotin's influence on lowering the degradation process. Furthermore, as shown in the figure, (304 L-alginate-HA) alloys displayed an increase in cell vitality with exposure time, showing that they have excellent compatibility and are suitable materials for implant fabrication. It also had no harmful impacts on human cells, enabling the study of a beneficial response to the alloy's surface. Because of the large changes in surface properties, it was clear that all samples' bioactivity did not develop correctly in response to cell viability. Because of variations in surface properties, the development of hydroxyapatite and biotin, which are essentially biomedical materials, did not increase surface bioactivity [32, 33].

## 5. Conclusions

The dip-coating deposition technique was used in synthesizing alginate-based composite coating reinforced with hydroxyapatite and biotin. Using different particle scale sizes as reinforcing material to alginate matrix aids in improving composite properties:

1. The FESEM images indicated homogeneous adhesion between the coating layer and the titanium, 304 stainless steel, and 316 L stainless steel substrate. This result confirmed the suitability of the proposed technique.
2. Contact angle results, the hydrophilicity of the surface decrease with the addition of hydroxyapatite or biotin. This behavior was due to the reinforcement effect, which promoted mechanical interlocking between the polymer matrix and particulate reinforcement, reduced roughness, and enhanced surface homogeneity.
3. MTT charts revealed that the treated alloys' surface became more reactive, leading to a rise in cell viability and cell attachment compared to untreated samples.
4. Addition of particles (hydroxyapatite, biotin) 10 Vol. percentage, this percentage was suitable to improve the properties of composite materials.

## Figures and Tables

**Table 1:** Samples coating composition on Ti substrate.

Sample no.	Composition of coating wt.%
1 2 3 4	100% pure Ti 90% alginate + 10% hydroxyapatite, 90% alginate + 10% biotin 90% alginate + 5% hydroxyapatite + 5% biotin

## Data Availability

The datasets used and/or analyzed during the current study available from the corresponding author on reasonable request.
